# A new aphelench nematode, *Basilaphelenchus brevistylus* n. sp. (Aphelenchoididae: Tylaphelenchinae) from *Pinus massoniana* in China

**DOI:** 10.21307/jofnem-2021-070

**Published:** 2021-08-05

**Authors:** Bashiy Akol, Qiuling Huang, Borong Lin, Honghong Wang, Jinling Liao, Kan Zhuo

**Affiliations:** 1 Laboratory of Plant Nematology, South China Agricultural University, Guangzhou, 510642, PR China; 2 Guangdong Laboratory of Lingnan Modern Agriculture, Guangzhou, 510642, PR China; 3 Guangdong Eco-Engineering Polytechnic, Guangzhou, 510520, PR China

**Keywords:** Aphelenchoididae, *Basilaphelenchus*, New species, Masson pine, Molecular phylogeny, Morphology, China

## Abstract

*Basilaphelenchus brevistylus* n. sp. was isolated from masson pine (*Pinus massoniana*) in Guangdong province, China. The new species is characterized by an offset lip region, short stylet (female stylet 4.5-5.5 μm and male stylet 4-5 μm long) with three elongate posteriorly directed knobs, posteriorly located metacorpal valve and lateral field composed of three lines. The female has an elongate postuterine sac and a short conical tail, uniformly narrowing toward a sharp tip, or tapering to a slightly offset mucronate tip in a few individuals. The male has a conical tail with a sharp terminal mucro, three pairs of caudal papillae, and small arcuate spicules with a bluntly rounded condylus and small pointed rostrum. *B. brevistylus* n. sp. can be distinguished from all described *Basilaphelenchus* nematodes by numerous morphological and morphometrical traits, especially the tail morphology of both sexes and stylet length. In addition, molecular phylogenetic trees inferred from rRNA small subunit and D2-D3 expansion domains of large subunit revealed that this nematode belongs to the *Basilaphelenchus*, and is clearly different from all the other *Basilaphelsenchus* species.

The family Aphelenchoididae Skarbilovich, 1947, with over 400 species, is a large group of aphelench nematodes (Hunt, 2008). Ecologically, they include phytoparasites, mycetophagous species, and predators. Many species are reported to be associates or parasites of insects (Hunt, 1993). Six subfamilies within the Aphelenchoididae were listed by Hunt (2008), whereas seven subfamilies were proposed on the basis of the classification for the Aphelenchoididae given by Kanzaki (2014). The difference between the two taxonomy systems is that the latter placed *Anomyctus* (Allen, 1940) in a separate subfamily, the Anomyctinae (Goodey, 1960). By the year of 2014, one new subfamily Tylaphelenchinae (Kanzaki et al., 2014) belonging to the Aphelenchoididae was established (Kanzaki et al., 2014). Currently four genera *Tylaphelenchus* (Rühm, 1956), *Pseudaphelenchus* (Kanzaki et al., 2009), *Albiziaphelenchus* (Bajaj, 2012), and *Basilaphelenchus* (Pedram et al., 2018) comprise the subfamily Tylaphelenchinae. Morphologically, they all have at least one tylenchid-like character, such as small spherical median bulb, tylenchid-type bursa, and elongate posteriorly directed stylet knobs (Mirzaie Fouladvand et al., 2019a). Phylogenetically, although molecular data are unavailable for the two genera *Tylaphelenchus* and *Albiziaphelenchus*, recent phylogenetic analysis based on rRNA small subunit (SSU) and D2-D3 expansion domains of large subunit (LSU D2-D3) confirmed that *Pseudaphelenchus* and *Basilaphelenchus* form a monophyly of the Tylaphelenchinae (Aliramaji et al., 2020; Kanzaki, 2021; Mirzaie Fouladvand et al., 2019a, b; Pedram et al., 2018).

*Basilaphelenchus*, the latest genus in Tylaphelenchinae, was erected in 2018. It currently contains seven species: *B. persicus* (Pedram et al., 2018); *B. grosmannae* (Pedram et al., 2018; Rühm, 1965); *B. gorganensis* (Mirzaie Fouladvand et al., 2019a); *B. brevicaudatus* (Mirzaie Fouladvand et al., 2019b); *B. magnabulbus* (Aliramaji et al., 2020); *B. pedrami* (Kanzaki, 2021), and *B. hyrcanus* (Golhasan et al., 2021). All *Basilaphelenchus* species are unique in stylet with three elongate and posteriorly directed knobs (Aliramaji et al., 2020; Kanzaki, 2021; Mirzaie Fouladvand et al., 2019a, b; Pedram et al., 2018; Rühm, 1965).

In a survey of aphelench nematodes from pine wood in China, an unknown species of aphelenchoidid was extracted from a dead *Pinus massoniana* Lamb. in Xingning city, Guangdong Province, China. Intensive morphological and molecular studies of the nematode revealed that it is a new species of the genus *Basilaphelenchus*. The new species is described and illustrated herein as *Basilaphelenchus brevistylus* n. sp. Phylogenetic analysis based on SSU and LSU D2-D3 was performed to investigate the relationships of the new species with other species of Tylaphelenchinae.

## Materials and methods

### Nematode extraction and morphological observations

Decaying wood and its bark samples were collected from a standing dead *Pinus massoniana* in Xingning city, Guangdong province in the south of China during early June 2020. The nematodes were extracted from samples by the Baermann funnel method (Feng, 2001), killed by gentle heat, fixed in DESS solution (Yoder et al., 2006), and processed by the glycerin-ethanol method for permanent slides (Seinhorst, 1959). Specimens were measured and photographed with the aid of a Nikon ECLIPSE Ni microscope equipped with a Nikon Digital Sight Camera and exclusive NIS-Elements BR software (Nikon, Tokyo, Japan).

### DNA extraction, amplification, and sequencing

DNA was extracted from three nematodes according to the protocol described in detail by Mundo-Ocampo et al. (2008). Two rRNA gene fragments, SSU and LSU D2-D3, were amplified. A combination of primers for SSU amplification were forward 1096F (5′-GGTAATTCTGGAGCTAATAC-3′) and reverse 1912R (5′-TTTACGGTCAGAACTAGGG-3′); forward 1813F (5′-CTGCGTGAGAGGTGAAAT-3′) and reverse 2646R (5′-GCTACCTTGTTACGACTTTT-3′) (Holterman et al., 2006). Primers for LSU D2-D3 amplification were forward D2A (5′-ACAAGTACCGTGAGGGAAAGTTG-3′) and reverse D3B (5′-TCGGAAGGAACCAGCTACTA-3′) (De Ley et al., 1999). PCR amplifications were performed according to the protocols as described previously (De Ley et al., 1999; Holterman et al., 2006). DNA fragments were sequenced as described by previous study (Zhuo et al., 2010). The newly obtained sequences of SSU and LSU D2-D3 were deposited in the GenBank database with accession numbers MW722958 and MW722960, respectively.

### Phylogenetic analysis

The sequences of *B. brevistylus* n. sp. were compared with aphelench nematode sequences in GenBank using the BLAST homology search program. The close-related and published sequences of aphelench nematodes were chosen for phylogenetic analyses. Outgroup taxa for each dataset were selected according to previous phylogenetic study for aphelench nematodes (Aliramaji et al., 2020). DNA sequences were aligned by ClustalW implemented in the program MEGA6.0 (Tamura et al., 2013) using default parameters. Models of base substitution were evaluated using Modeltest3.7 (Posada and Crandall, 1998) combined with PAUP4.0 (Swofford, 1998). The Akaike-supported model, the base frequencies, the proportion of invariable sites, the gamma distribution shape parameters, and substitution rates were used in our phylogenetic analyses. Bayesian analysis for SSU and LSU D2-D3 under the GTR + I + G model was employed to confirm the tree topology using MrBayes 3.2 (Huelsenbeck and Ronquist, 2001) running four chains for 1 × 10^6^ generations and setting the ‘burn-in’ at 2,500. The MCMC (Markov Chain Monte Carlo) method was used within a Bayesian framework to estimate the posterior probabilities of the phylogenetic trees (Larget and Simon, 1999) and generate a 50% majority rule consensus tree. TreeView1.6 was used to display and edit the trees (Page, 1996).

## Results

### Systematics

*Basilaphelenchus brevistylus* n. sp.

(Figs. 1-3).

**Figure 1:**
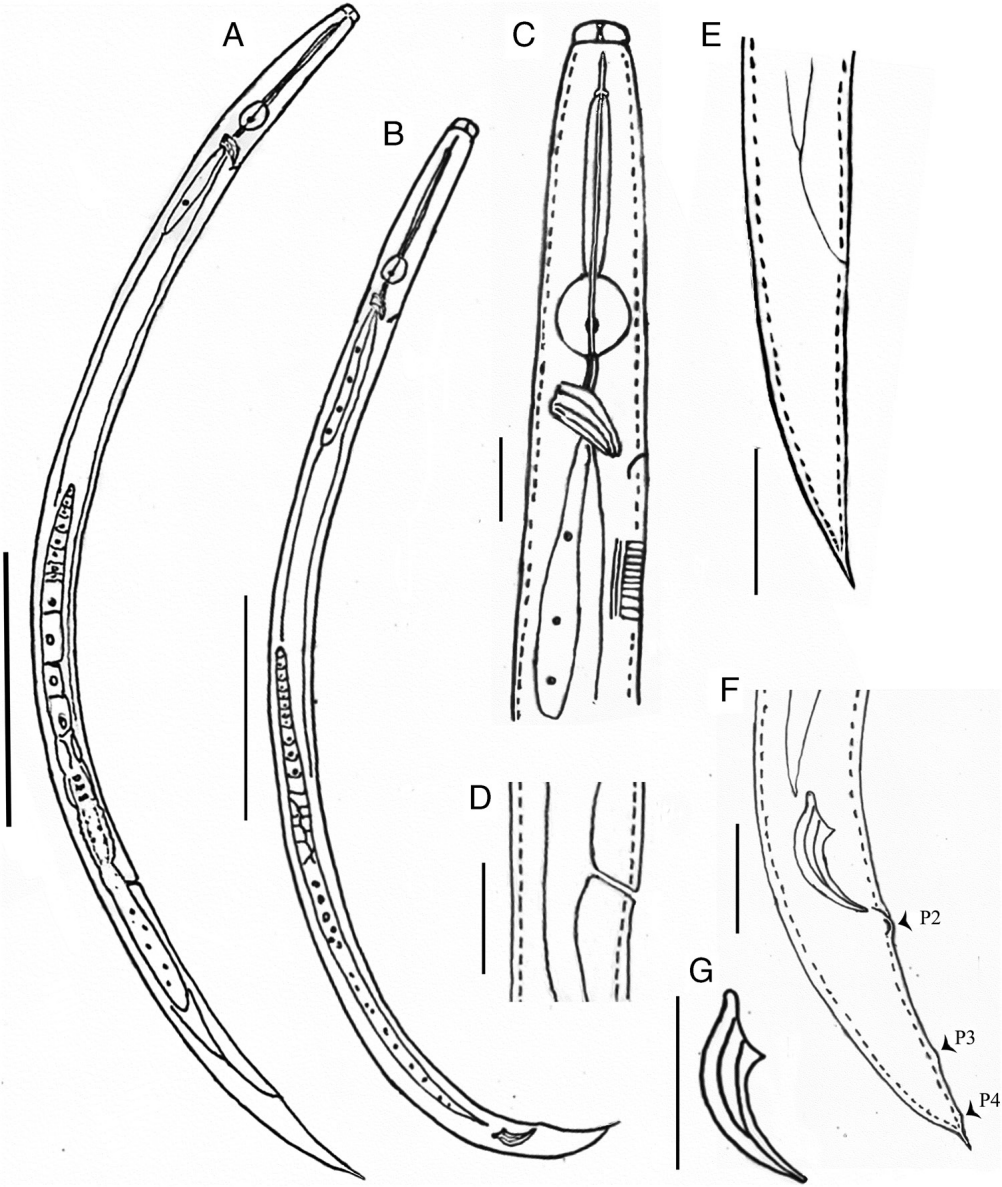
Line drawings of *Basilaphelenchus brevistylus* n. sp. (A) female; (B) male; (C) female anterior region; (D) vulval region; (E) female tail; (F) male tail; (G) spicule. (Scale bars: A, B = 50 µm; C-G = 10 µm).

**Figure 2:**
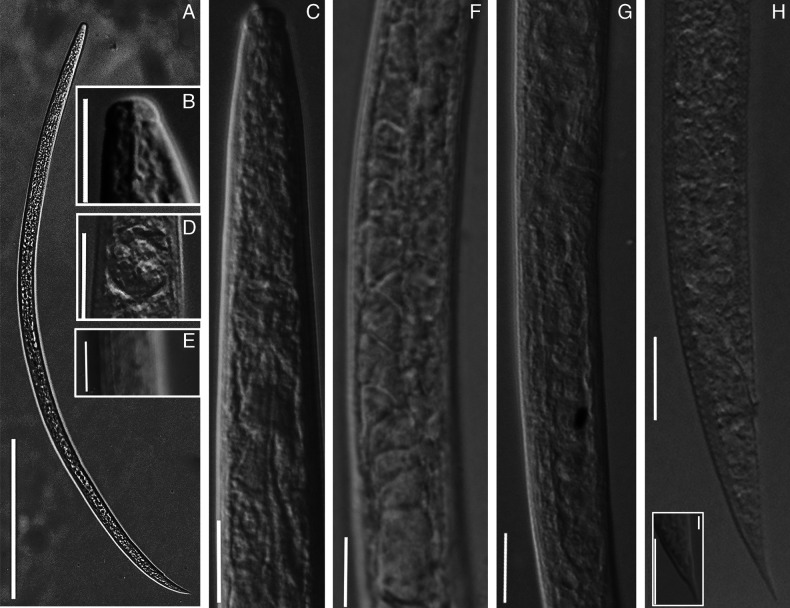
Females of *Basilaphelenchus brevistylus* n. sp. under the light microscrope. (A) entire body; (B) lip region and stylet; (C) anterior region; (D) metacorpus; (E) lateral lines; (F) oocytes; (G) vulva region and post-vulval sac; (H) tail. (Scale bars: A = 100 µm; B-H = 10 µm).

**Figure 3:**
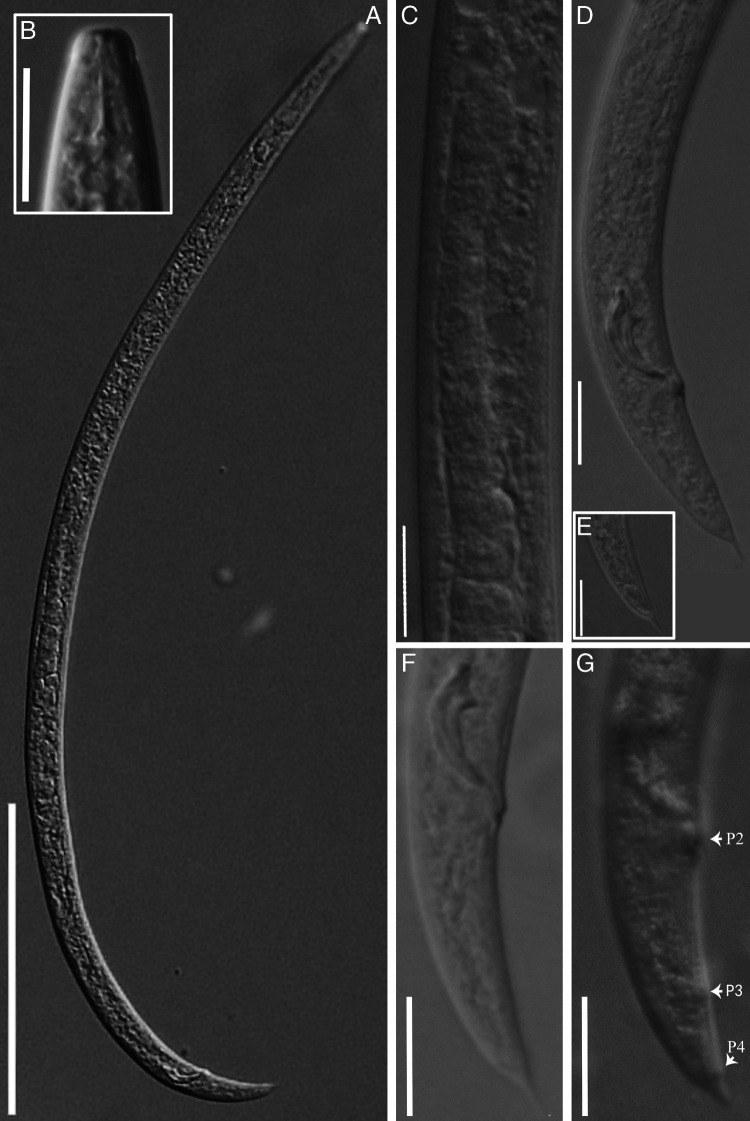
Males of *Basilaphelenchus brevistylus* n. sp. under the light microscrope. (A) entire body; (B) lip region and stylet; (C) testis cells; (D, F) tail and spicule; (E) tail tip; (G) papillae. (Scale bars: A = 100 µm; B-G = 10 µm).

#### Measurements

Measurements of the new species are given in Table 1.

**Table 1.** Morphometrics of *Basilaphelenchus brevistylus* n. sp

	Female		Male
Character	Holotype	Paratypes	Paratypes
*n*	–	15	15
*L*	469.1	413.6 ± 35.4 (364.3-483.1)	381.7 ± 19.6 (352.4-413.1)
*a*	29.7	29.2 ± 2.9 (25.2-36.8)	33.6 ± 3.0 (29.6-40.5)
*b*	10.4	9.9 ± 0.5 (9.4-10.9)	8.9 ± 0.5 (8-9.6)
*c*	18.5	17.7 ± 1.3 (16.2-20.9)	16.2 ± 1.0 (13.9-17.8)
*c′*	3.0	3.1 ± 0.3 (2.7-3.5)	2.9 ± 0.2 (2.6-3.4)
*V* or *T*	72.2	71.7 ± 1.1 (69.8-73.8)	49.9 ± 4.6 (43.2-61.7)
*M*	30.0	32.5 ± 3.7 (28.0-38.0)	31.0 ± 4.4 (24.0-37.0)
Max. body diam.	15.8	14.1 ± 1.8 (11.4-18.0)	11.4 ± 1.1 (9.8-13.2)
Lip region diam.	4.4	4.5 ± 0.4 (4.1-5.3)	4.4 ± 0.2 (4-4.8)
Lip region height	2.5	2.4 ± 0.3 (2-2.6)	2.3 ± 0.1 (2.1-2.5)
Stylet conus	1.5	1.6 ± 0.2 (1.4-1.9)	1.4 ± 0.2 (1.1-1.8)
Stylet length (total)	5.0	4.9 ± 0.3 (4.5-5.5)	4.6 ± 0.4 (4.0-5.0)
Distal end of metacorpus from anterior	45.0	41.5 ± 2.5 (37.7-45.1)	42.6 ± 1.3 (39.0-43.7)
Metacorpus length	9.2	8.4 ± 0.6 (7.3-9.5)	8.3 ± 0.6 (7.3-9.7)
Metacorpus diam.	6.6	6.8 ± 0.7 (5.8-8.1)	6.6 ± 0.5 (5.4-7.8)
Position of the valve of metacorpus (%)	65.2	66.0 ± 3.7 (60.7-72)	67.3 ± 2.7 (63-72)
Body diam. at metacorpus level	10.9	11.0 ± 2.4 (9.7-11.7)	9.6 ± 0.7 (8.5-10.8)
Gonad length	213.3	190.1 ± 21.8 (160.1-223.9)	189.9 ± 18.4 (163.9-242.6)
Vulval body diam.	12.0	12.1 ± 1.2 (10.5-14.4)	–
Spicule length (chord)	–	–	11.5 ± 0.7 (10.3-12.8)
Spicule length (arc)	–	–	10.5 ± 0.6 (9.6-11.3)
Post-vulval uterine sac length	48.8	45.9 ± 7.0 (36.6-56.5)	–
Vulva-anus distance	104.9	93.7 ± 7.1 (83.1-107.3)	–
Anal or cloacal body diam.	8.4	7.3 ± 0.8 (6.5-8.9)	8.0 ± 0.3 (7.5-8.7)
Tail length	25.4	22.0 ± 0.6 (20-26.6)	23.5 ± 1.6 (20.1-27.3)

**Notes:** All measurements are in µm and in the form: mean ± s.d. (range).

#### Description

##### Female

Small size. Body slender and slightly ventrally curved when heat-relaxed; annules fine. Lateral fields with three incisures. Lip region raised, 1.5 to 2.5 times wider than high, offset from body, separated from body by a clear constriction; vestibule well sclerotized, X-shaped in lateral view. Stylet short, 4.5 to 5.5 μm long, with three elongate and posteriorly directed knobs, stylet cone comprising *ca.* 30% of total stylet. Procorpus cylindrical, *ca*. three to four stylet lengths. Metacorpus (median bulb) small, spherical, its width 66.5 ± 3.2 (59.1-78.3)% corresponding body diam., with glandular anterior part and muscular posteior part. Valve of median bulb weak, but discernible, situating posteriorly, at 60.7 to 72.0% of metacorpus length from anterior end of metacorpus. Pharyngo-intestinal junction immediately posterior to metacorpus. Nerve ring encircling intestine and pharyngeal glands, and *ca.* 1/4-3/4 metacorpal length posterior to metacorpus. Excretory pore usually difficult to observe, posterior to metacorpus, at the level of nerve ring. Hemizonid invisible. Pharyngeal glands overlapping intestine dorsally for *ca.* 2.5 to 3.5 body diameters. Three glands observed, each containing a nucleus separately. Reproductive system monodelphic, outstretched, occupying 38.9 to 61.7% of body length (excluding post-vulval uterine sac), oocytes present in single row; oviduct connecting ovary and spermatheca; spermatheca elongate-oval, sperms present in some individuals; crustaformeria ovate-oblong, posterior to spermatheca, visible in some individuals; uterus with thick wall, posterior to crustaformeria. Vagina inclined anteriorly at *ca.* 45° to body axis, both anterior and posterior vulval lips slightly protruding, vulval flap absent. Postuterine sac long, 36.2 to 56.5 μm long, extending 40.0 to 62.5% of vulva-anus distance, *ca.* 2.6 to 4.9 vulval body widths or 4.2 to 8.0 anal body widths long, sperms usually present. Intestine simple, rectum and anus functional. Tail conical, short, uniformly narrowing toward a sharp tip, or tapering to a slightly offset mucronate tip in a few individuals.

##### Male

About equal number as females. Body slender and slightly ventrally curved when heat-relaxed. Anterior region and cuticle similar to female. Testis single, cells in single row, anteriorly outstretched, occupying 43.3 to 61.7% of body length. Spicules paired, separate, condylus bluntly rounded, rostrum small and pointed, capitulum with shallow depression, calomus-lamina complex (blade) smoothly tapering and smoothly ventrally curving to a fine rounded terminus, cucullus not observed. Gubernaculum absent. Three pairs of papilliform caudal papillae, i.e., P2 subventral adcloacal, P3 post and near the middle of tail, and P4 near to tail tip. Tail short, conoid, with a sharp terminal mucro, *ca.* 2.5 to 3.5 μm.

##### Type host and locality

The type material was isolated from *Pinus massoniana* in Xingning city (latitude N23°98.753′, longitude E115°91.007′), Guangdong province, PR China in June 2020.

##### Type specimens

The holotype female, 15 female and 15 male paratypes are deposited in Laboratory of Plant Nematology, College of Plant Protection, South China Agricultural University, Guangzhou, China. Five paratype females and five paratype males are deposited in the USDA Nematode Collection, Beltsville, MA, USA.

##### Etymology

The specific epithet is derived from the shorter stylet of the new species compared with the other *Basilaphelenchus* species.

##### Differential diagnosis

Except the general characteristics of the genus *Basilaphelenchus*, i.e. stylet having three elongate posteriorly directed knobs and posteriorly located valve of metacorpus (median bulb), *Basilaphelenchus brevistylus* n. sp. is also characterized by three incisures in the lateral field, an offset lip region, very short stylet (4.5-5.5 μm in females and 4-5 μm in males), long postuterine sac (extending *ca*. 40.0-62.5% of vulva-anus distance), short conical tail of both sexes, female tail narrowing toward a sharp tip or tapering to a slightly offset mucronate tip in a few individuals, male tail bearing a sharp terminal mucro, and small arcuate spicules with a bluntly rounded condylus and small pointed rostrum. And the new species has specific LSU D2-D3 and SSU sequences.

Currently seven *Basilaphelenchus* species have been reported. *B. brevistylus* n. sp. can be distinguished from these seven species by the tail shape of both sexes (terminus not bent ventrally vs obviously or strongly bent ventrally) and a shorter stylet (4.5-5.5 vs 5-10 μm in females and 4-5 vs 5-10 μm in males). Besides these, the new species differs from *B. grosmannae* by the different spicule shape (blade smoothly curved vs blade somewhat straight), more anteriorly located vulva (*V* = 71.7 (69.8-73.8) vs 72.9-75.9), a higher *b* ratio (9.9 (9.4-10.9) vs 6.5-7.1 in females and 8.9 (8-9.6) vs 6.3-6.4 in males) and smaller cloacal body diam. (8 (7.5-8.7) vs 11.6 μm); from *B. persicus* by the more posteriorly located vulva (*V* = 71.7 (69.8-73.8) vs 65.7 (63.6-70.8)), a longer body (413.6 (364.3-483.1) vs 352 (297-393) μm in females and 381.7 (352.4-413.1) vs 322 (304-331) μm in males), shorter postuterine sac (45.9 (36.6-56.5) vs 62 (50-70) μm), shorter tail of females (22 (20-26.6) vs 36 (29-45) μ m; *c* = 17.7 (16.2-20.9) vs 9.7 (8.3-11.8); *c′* = 3.1 (2.7-3.5) vs 5.3 (4.1-6.7)) and males (23.5 (20.1-27.3) vs 30 (24.5-36) μm; *c* = 16.2 (13.9-17.8) vs 10.7 (9-13); *c′* = 2.9 (2.6-3.4) vs 3.9 (3.2-4.6)), and higher *b* ratio (9.9 (9.4-10.9) vs 7.4 (6.9-8) in females and 8.9 (8-9.6) vs 6.3 (5-7) in males); from *B. gorganensis* by a shorter postuterine sac (45.9 (36.6-56.5) vs 68 (59-79) μm; extending for 40.0-62.5% vs. 60-70% of vulva-anus distance) and lower *a* ratio (29.2 (25.2-36.8) vs 40 (34.2-47.7) in females; 33.6 (29.6-40.5) vs 41.7 (36.3-52.5) in males); from *B. brevicaudatus* by the different tail tip shape of females (sharp vs generally broadly rounded, rarely narrow, sometimes with a mucron), a longer postuterine sac (45.9 (36.6-56.5) vs 32.4 (29-37) μm; extending for 40.0-62.5% vs 30-40% of vulva-anus distance), lower *c* ratio of females (17.7 (16.2-20.9) vs 22.5 (19.5-26.6)) and males (16.2 (13.9-17.8) vs 19.3 (18-21)), lower *a* ratio of females (29.2 (25.2-36.8) vs 37.2 (33.8-44.2)) and males (33.6 (29.6-40.5) vs 43 (38-49)) and shorter spicule chord (11.5 (10.3-12.8) vs14 (13-15) μm); from *B. magnabulbus* by the different male spicule shape (relatively obvious capitulum depression vs somewhat straight capitulum anterior surface), different tail tip shape of males (terminus with a sharp mucro *vs* bluntly to finely rounded), a shorter female tail (22 (20-26.6) vs 26-46 μm; *c* = 17.7 (16.2-20.9) vs 9.9-13; *c′* = 3.1 (2.7-3.5) vs 6-9.3), longer male tail (23.5 (20.1-27.3) vs 14-19 μ m; *c* = 16.2 (13.9-17.8) vs 17.3-43.9; *c′* = 2.9 (2.6-3.4) vs 1.6-2.5), lower female *a* ratio (29.2 (25.2-36.8) vs 40.6-53.9) and higher male *b* ratio (8.9 (8-9.6) vs 6-7.9); from *B. pedrami* by the different tail tip shape of males (tip with a long and sharp mucro *vs* tip with a short and blunt mucro), a shorter female tail (22 (20-26.6) vs 28 (25-32) μm; *c* = 17.7 (16.2-20.9) vs 15.4 (14.7-16.7); *c′* = 3.1 (2.7-3.5) vs 4.3 (3.7-4.9)), lower female *a* ratio (29.2 (25.2-36.8) vs 36.8 (35-38.4)) and higher *b* ratio (9.9 (9.4-10.9) vs 9 (8.3-9.8) in females and 8.9 (8-9.6) vs 7.8 (7.1-8.2) in males); from B. *hyrcanus* by a shorter tail of females (22 (20-26.6) vs 30 (28-31) μm; *c* = 17.7 (16.2-20.9) vs 13 (11.5-14); *c′* = 3.1 (2.7-3.5) vs 5.4 (4.8-6.2)) and males (23.5 (20.1-27.3) vs 24.5-36 μm; *c* = 13.9-17.8 vs 9-13; *c′* = 2.6-3.4 vs 3.2-4.6), higher *b* ratio (9.9 (9.4-10.9) vs 8.3 (7.3-9.5) in females and 8.9 (8-9.6) vs 7.3 (7-7.6 in males), more rounded median bulb (8.4 (7.3-9.5) × 6.8 (5.8-8.1) vs 10.7 (10-12) × 6.2 (5-7) μm in females and 8.3 (7.3-9.7) × 6.6 (5.4-7.8) vs 11.1 (11-12) × 6.3 (6-7) μm in males), different tail tip shape of males (terminus with a sharp mucro *vs* sharp or finely rounded tip or small mucron like projection) and smaller anal body diam. (7.3 (6.5-8.9) vs 5.6 (5-6) μm).

In addition, the SSU phylogenetic analysis revealed that *B. brevistylus* n. sp. has a close relationship to *Pseudaphelenchus* spp., however, *B. brevistylus* n. sp. can be easily distinguished from *Pseudaphelenchus* spp. by the absence or presence of bursa (male tail without bursa vs male tail with long bursa), the different stylet shape (stylet with three elongate posteriorly directed knobs vs stylet bipartite with small and conspicuous basal knobs) and a shorter body of female (less than 500 μm vs generally more than 500 μm).

##### Molecular profiles and phylogenetic status

**Figure 4:**
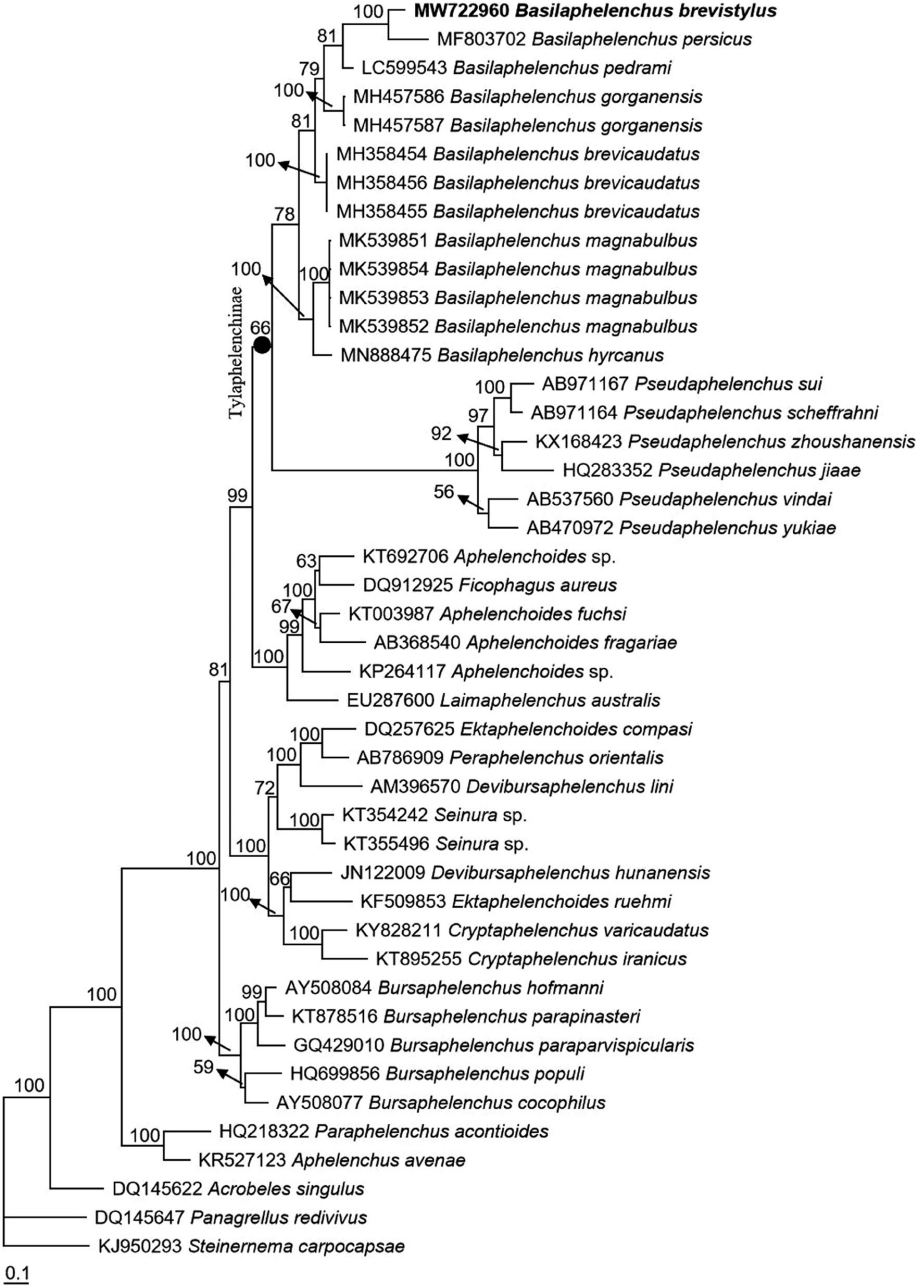
Bayesian consensus tree inferred from D2-D3 under GTR + I + G model (‒lnL = 18,080.8008; freqA = 0.1951; freqC = 0.1764; freqG = 0.3257; freqT = 0.3028; R(a) = 0.8366; R(b) = 2.5599; R(c) = 1.221; R(d) = 0.5867; R(e) = 4.2157; R(f) = 1; Pinva = 0.1777; Shape = 0.9323). Posterior probability values exceeding 50% are given on appropriate clades.

**Figure 5:**
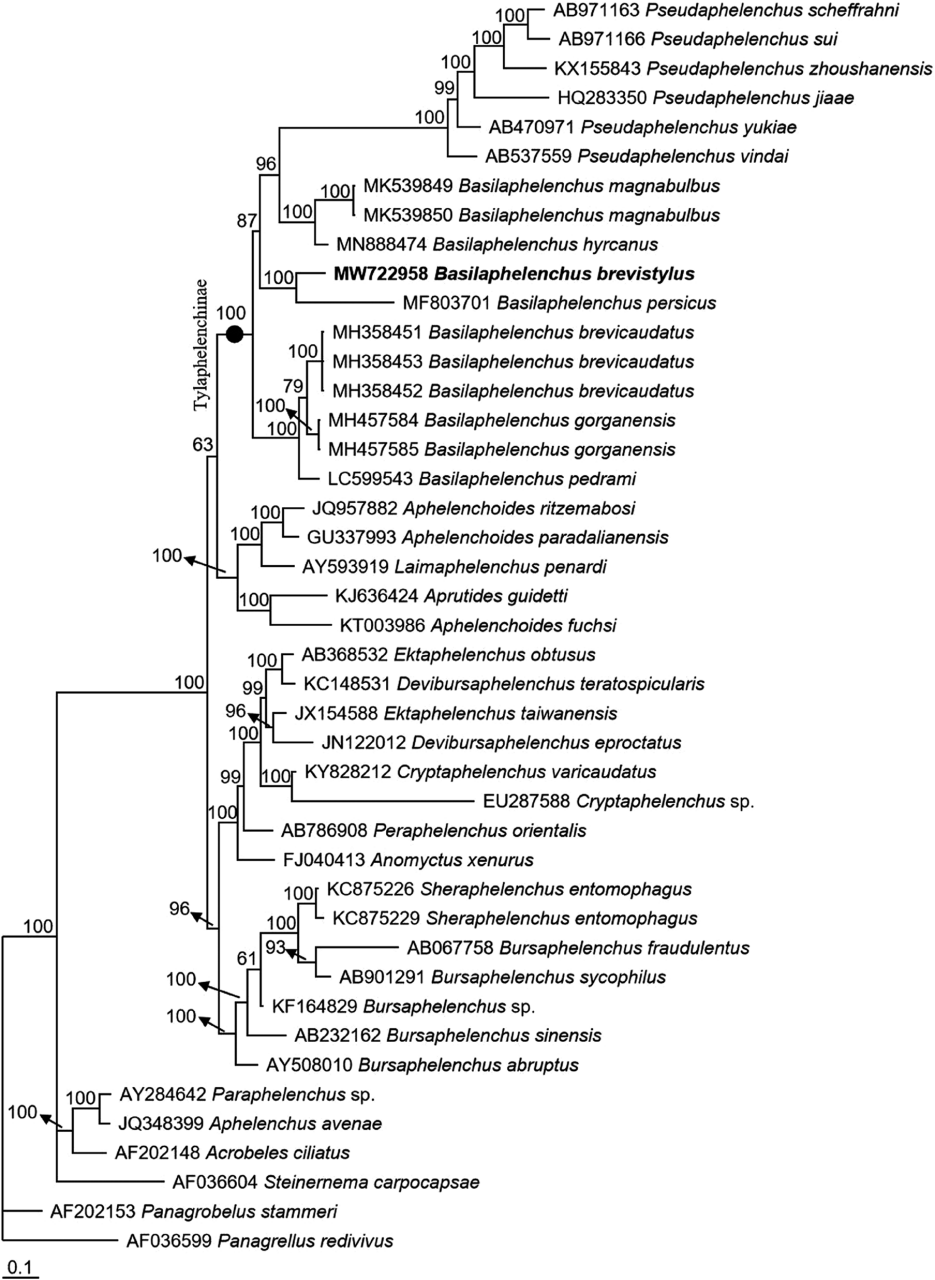
Bayesian consensus tree inferred from SSU under GTR + I + G model (‒lnL = 27,834.9805; freqA = 0.2254; freqC = 0.1985; freqG = 0.2797; freqT = 0.2964; R(a) = 1.0461; R(b) = 2.3739; R(c) = 1.4729; R(d) = 0.7716; R(e) = 2.9919; R(f) = 1; Pinva = 0.0959; Shape = 0.7961). Posterior probability values exceeding 50% are given on appropriate clades.

The 628-bp LSU D2-D3 and 1597-bp near full-length SSU were sequenced. The molecular phylogenetic status of *B. brevistylus* n. sp. is presented in Figures 4 and 5, and the two phylogenetic trees reconstructed based on sequences of LSU D2-D3 and SSU both confirm that the new species was within the *Basilaphelenchus* clade. In Figure 4, the phylogenetic tree is based on LSU D2-D3 from a multiple alignment of 1142 total characters, all *Basilaphelenchus* species reside within a 78% supported monophyletic clade. In the clade, *B. brevistylus* n. sp. is closely related to *B. persicus* with a 100% support, and they are clearly distinguished from each other. And the *Basilaphelenchus* clade is sister to the *Pseudaphelenchus* clade, forming a monophyletic clade of Tylaphelenchinae with a 66% support. In Figure 5, the phylogenetic tree is based on SSU from a multiple alignment of 2,734 total characters, *B. brevistylus* n. sp. is also closely related to *B. persicus* with a 100% support and clearly distinguished from it, but these two species and other *Basilaphelenchus* species do not form a monophyletic clade. Similar with the tree inferred from LSU D2-D3, all *Basilaphelenchus* and *Pseudaphelenchus* species form a monophyly of the subfamily Tylaphelenchinae, with a 100% support.

## Discussion

In China, the genus *Basilaphelenchus* has not been reported to date. The finding of *Basilaphelenchus brevistylus* n. sp. expands the geographic distribution of this genus. The *Basilaphelenchus* is a relatively new genus within the family Aphelenchoididae. It was established in 2018 (Pedram et al., 2018). Since then, six *Basilaphelenchus* have been reported. Five of the six were found in Iran (Aliramaji et al., 2020; Golhasan et al., 2021; Mirzaie Fouladvand et al., 2019a, b; Pedram et al., 2018), and the remaining one was described in Japan more recently (Kanzaki, 2021). Besides, *Tylaphelenchus grosmannae* (Rühm, 1965), originating from Chile, was transferred to the genus *Basilaphelenchus* as *B. grosmannae* due to typological similarities (Pedram et al., 2018). Therefore, *B. brevistylus* n. sp. is the eighth *Basilaphelenchus* species. So far, all *Basilaphelenchus* species were found in wood of trees, including *Araucaria araucana*, *Fagus orientalis*, and several unidentified trees (Golhasan et al., 2021; Kanzaki, 2021). In this study, *B. brevistylus* n. sp. was isolated from *Pinusm massoniana*, which is the first report of the genus from pine tree.

Currently, little is known about the biology of the genus *Basilaphelenchus.* However, a mycetophagus habit for this genus has been suggested as all *Basilaphelenchus* species were found in dead wood and rotten material, and multiple species, including *B. persicus*, *B. pedrami*, *B. hyrcanus*, and *B. gorganensis*, had been successfully multiplied on fungi (Golhasan et al., 2021; Kanzaki, 2021). Although we did not try to culture *B. brevistylus* n. sp. in fungi, the new species was also extracted from decaying wood. We therefore agree with the mycophagy hypothesis for this genus. In addition, it has also been proposed that this genus may be associated with wood borer and bark beetle insects because all *Basilaphelenchus* species were from wood and bark samples (Golhasan et al., 2021). However, so far only *B. grosmannae* was discovered to be carried by a bark beetle *Hylurgonotus brunneus* (Rühm, 1965). Insect associations of the other seven *Basilaphlenchus* species including *B. brevistylus* n. sp. have not been demonstrated. Interestingly, we noted that all *Basilaphelenchus* species stylets have an unique shape (with three elongate and posteriorly directed knobs) and are very short (no more than 10 μ m). It has been found that stylet shape and length of several aphelenchoidid species are related to their biological characters. For example, *Bursaphelenchus sinensis* showed morphological differences between a mycophagous and predaceous form (Kanzaki et al., 2019); the parasitic generation of *Bursaphelenchus sexdentati* has a smaller stylet than the free-living generation (Vosilite, 1990). Therefore, it would be valuable to further investigate potential insect carriers of *Basilaphelenchus* nematodes and the possible stylet modifications indicative of a specific insect-nematode relationship.

Given that the small body sizes and morphological similarity with *Aphelenchoides*, it is possible that the *Basilaphelenchus* nematodes were overlooked during nematode surveys, and molecular techniques are of great assistance to confirm the status of *Basilaphelenchus* spp. (Kanzaki, 2021). In this study, our molecular phylogenetic analyses based on two rDNA markers, LSU D2-D3 and SSU, both place *B. brevistylus* n. sp. in a highly supported clade with *B. persicus*, and *B. brevistylus* n. sp. is clearly distinguished from all the other *Basilaphelenchus* species, which is in line with the result of morphological identification, confirming this nematode is a new *Basilaphelenchus* species. Interestingly, the paraphyly of the genus *Basilaphelenchus* had been indicated according to several studies based on phylogenetic analyses inferred from SSU and LSU D2-D3, and *Basilaphelenchus* and *Pseudaphelenchus* always formed a Tylaphelenchinae monophyly (Aliramaji et al., 2020; Kanzaki, 2021; Mirzaie Fouladvand et al., 2019a, b). In our study, *Basilaphelenchus* spp. are closely related to *Pseudaphelenchus* spp. but the exact nature of that relationship is not clear. Both LSU D2-D3 and SSU provide weak to moderate support for a sister genus relationship and the monophyly of *Basilaphelenchus*. To date we have not identified a consistent morphological or host-range character that define the relevant clades. Similar unresolved relationships have been reported in other Tylenchina, e.g. *Rotylenchus* (Cantalapiedra-Navarretea et al., 2013), *Filenchus* (Qing et al., 2017), *Mesocriconema*, and *Criconemoides* (Powers et al., 2017). We believe additional genetic markers and additional taxa will improve our understanding of the relationships in this often overlooked genus of fungal feeding nematodes.
